# Relationship between child-to-parent violence and cumulative childhood adversity: the mediating role of parental attachment, resilience, and emotional intelligence

**DOI:** 10.3389/fpsyg.2023.1135419

**Published:** 2023-05-31

**Authors:** María J. Navas-Martínez, M. Carmen Cano-Lozano

**Affiliations:** Department of Psychology, University of Jaén, Jaén, Spain

**Keywords:** adolescents, aggressors, profiles, child-to-parent violence, cumulative childhood adversity, parental attachment, resilience, emotional intelligence

## Abstract

**Introduction:**

Recent research on aggressor profiles in child-to-parent violence (CPV) seems to provide promising results. However, this phenomenon has been poorly addressed in the adverse childhood experiences (ACEs) framework. This study aimed to explore the frequency of different types of ACEs and cumulative ACEs in adolescents who exert CPV, to analyze the differences between aggressors with different levels of cumulative ACEs in parental attachment, resilience, and emotional intelligence, and to evaluate the associations between these variables, as well as a possible mediational model.

**Methods:**

A total of 3,142 Spanish adolescents (50.7% girls) aged between 12 and 18 years from educational centers participated.

**Results:**

Adolescents who exerted CPV presented higher rates of ACEs both independently and cumulatively than those without CPV. Aggressors with cumulative ACEs (88%) in general presented more insecure parental attachment, lower resilience, and lower emotional intelligence than those without cumulative ACEs, and, in turn, aggressors with high levels of cumulative ACEs than those with low levels of cumulative ACEs. Significant associations were identified between CPV, ACEs, insecure parental attachment, resilience, and emotional intelligence. The mediation model suggested that ACEs are related to CPV through preoccupied and traumatized parental attachment and also through low levels of emotional intelligence.

**Discussion:**

The findings provide a better understanding of CPV from the perspective of ACEs, especially of those cases that involve an accumulation of adverse experiences during childhood, and suggest greater professional attention to these cases with the design of specialized CPV intervention programs.

## 1. Introduction

Child-to-parent violence (CPV) is a type of family violence in which the children show a set of behaviors of psychological, physical, and/or financial violence to gain power, control (Cottrell, 2001), and domain over parents (Howard and Rottem, 2008), consciously, intentionally and repeatedly over time (Molla-Esparza and Aroca-Montolío, 2018). International research shows that it is an increasingly frequent psychosocial phenomenon with a very negative impact on the health of the children and parents involved (Ibabe, 2020). The magnitude varies depending on the type of CPV. In general terms, psychological violence ranges between 28.8 and 91.5%, physical violence ranges between 2.5 and 25%, and financial violence and control and domain behaviors are around 60 and 70%, respectively (Margolin and Baucom, 2014; Beckmann et al., 2021; Cano-Lozano et al., 2021a).

Numerous studies have analyzed the characteristics associated with the family, individual and social contexts of CPV aggressors (see Simmons et al., 2018, for review). However, few studies have explored this phenomenon from the framework of adverse childhood experiences (ACEs; Nowakowski-Sims and Rowe, 2017; Nowakowski-Sims, 2019; Navas-Martínez and Cano-Lozano, 2022a,b). ACEs are potentially traumatic events that occur before the age of 18, ranging from victimization experiences of diverse types (Finkelhor et al., 2007, 2013; World Health Organization, 2018) to a variety of other serious incidents and household dysfunctions (World Health Organization, 2018). Victimization experiences refer mainly to the abuse and neglect that a child or adolescent receives from other social agents, such as parents (e.g., direct abuse by parents, abandonment by parents, and witnessing of abuse between parents) and peers (e.g., direct abuse and cyber-abuse by peers), while the other types of ACEs refer, for example, to exposure to accidents, serious diseases, protection centers, or growing up in a family environment marked by incarceration, drug use, psychological problems, and/or suicide of family members.

ACEs have been consistently related to a greater risk of internalizing problems (e.g., depression or anxiety; Chapman et al., 2004) and externalizing problems (e.g., violence or delinquency; Ford et al., 2012) as a consequence of the traumatic damage suffered. Moreover, the risk of these problems seems to be even greater in those cases in which multiple types of ACEs occur simultaneously (Finkelhor et al., 2007, 2013; Bryce and Collier, 2022), in other words, when there is cumulative childhood adversity. In the field of CPV, numerous studies have found that both direct and witnessing parental abuse are powerful predictors of CPV (e.g., Calvete et al., 2014; Margolin and Baucom, 2014; Contreras and Cano-Lozano, 2016; Beckmann, 2019; Contreras et al., 2020; Ibabe et al., 2020; Simmons et al., 2020; Cano-Lozano et al., 2021b; Navas-Martínez and Cano-Lozano, 2022a), increasing the probability of CPV up to 70% (see Gallego et al., 2019, for review). More recently, it has been found that experiences of peer victimization, both direct and cyber peer abuse, also predict the development of CPV (Beckmann, 2019; Navas-Martínez and Cano-Lozano, 2022a). Furthermore, in line with the general research on cumulative ACEs, a study identified that the simultaneous presence of parental and peer ACEs predicted CPV to a greater extent than the separate presence of these two types of ACEs (Navas-Martínez and Cano-Lozano, 2022a).

Regarding frequency, between 68.2 and 83% of the adolescents in the general population have experienced at least one ACE in their lives (Copeland et al., 2007; Finkelhor et al., 2007; Pereda et al., 2014). Concerning CPV and ACEs related to parental victimization, it has been observed that adolescents who exert this type of violence present higher levels of direct and witnessing parental abuse compared to those who do not exert it (Contreras and Cano-Lozano, 2016; Simmons et al., 2020; Martín et al., 2022). Specifically, 25% of adolescents with CPV offenses suffer direct parental abuse and 54% are witnesses of parental abuse between parents (Nowakowski-Sims and Rowe, 2017), whereas 32.5% of CPV aggressors experience some of these two types of abuse repeatedly (Navas-Martínez and Cano-Lozano, 2022b), and 48% also inform having an uninvolved parent (Nowakowski-Sims and Rowe, 2017). Some authors have also found more ACEs related to peer victimization in CPV aggressors than in non-aggressors (Contreras and Cano-Lozano, 2016), whereas others have not (Martín et al., 2022). Nevertheless, Calvete et al. (2015a) pointed out that most adolescents with CPV offenses suffered direct peer abuse, according to the information of their parents. More recently, it has been found that 8.5% of CPV aggressors experienced direct or cyber peer abuse repeatedly (Navas-Martínez and Cano-Lozano, 2022b). Concerning the other types of ACEs, only Nowakowski-Sims and Rowe (2017) examined some of them and found that 13% of adolescents with CPV offenses had a serious accident, whereas drug use, psychological problems, and imprisonment by parents were informed with frequency of 14, 17, and 42%, respectively.

The simultaneous frequency of two or more ACEs (or cumulative ACEs) occurs in 37–69% of adolescents of the general population (Copeland et al., 2007; Finkelhor et al., 2007), whereas adolescents with CPV offenses experience an accumulation of 10 out of 17 ACEs on average (Nowakowski-Sims and Rowe, 2017). Focusing more specifically on the cumulative proportion of the most researched ACEs, that is victimization experiences, 22% of children and adolescents of the general population experienced multiple types of victimization (Finkelhor et al., 2007), which is close to 19.3% found in Spanish adolescents (Pereda et al., 2014). Regarding CPV, 22% of Spanish adolescents who exercised CPV suffered parental and peer abuse repeatedly (Navas-Martínez and Cano-Lozano, 2022b).

Previous literature suggests that ACEs tend to co-occur and that this accumulation seems to be frequent in cases of CPV. Moreover, it is observed that the cumulative effect of different types of ACEs further increases the risk of CPV. However, no study has provided, to date, the frequency of all the ACEs reviewed and their co-occurrence in cases of CPV. Likewise, it has not been examined whether these experiences are more frequent in adolescents who exert CPV than in those who do not involve in this type of violence, which provides a partial understanding of these phenomena.

On the other hand, part of the most recent research on CPV bets on the study of aggressors’ profiles (Moulds et al., 2019; Nowakowski-Sims, 2019; Martínez-Ferrer et al., 2020; Vecina et al., 2020; Boxall and Sabol, 2021; Navas-Martínez and Cano-Lozano, 2022b,c, 2023; Cano-Lozano et al., 2023), by finding different characteristics among aggressors classified into different typologies. The results of these studies suggest that different types of aggressors demand different types of interventions. Navas-Martínez and Cano-Lozano (2022b) identified an aggressor profile specifically focused on the ACEs related to parental and peer abuse. They found that the aggressors who experienced only parental abuse and only peer abuse showed more insecure parental attachment styles and lower resilience and emotional intelligence than aggressors without abuse experiences; in turn, the same was found for the aggressors who experienced an accumulation of both types of abuse compared to those who experienced only one type of abuse. Given the apparent co-occurrence of ACEs in cases of CPV, analyzing the characteristics of the aggressors classified as a function of the level of cumulative ACEs (Finkelhor et al., 2007; Pereda et al., 2014) could also be useful for research and professional practice by identifying the most affected areas in each case. Furthermore, since previous studies have found associations between different ACEs and the risk of CPV, it would be convenient to explore possible explanatory mechanisms for the relationship between ACEs and CPV.

In relation to the aforementioned, the literature suggests that childhood adversity has a series of negative effects on children’s developmental evolution. For example, it has been found that ACEs are related to insecure parental attachment and lower levels of resilience and emotional intelligence (Arslan, 2016; Lätsch et al., 2017; Huang and Mossige, 2018; Barnett and Howe, 2021) and predicts insecure parental attachment style as well as emotional, adaptive, and behavioral problems in the child (see Bryce and Collier, 2022, for review). These studies have also found correlations between attachment, resilience, and emotional intelligence, which are variables that could be included in attachment theory (Bowlby, 1979). This theory has been frequently used to explain the effects of ACEs in evolutionary development and defines attachment as a mental representation of the affective bond established from the interaction between the child and the attachment figure in the first years of life. Depending on the availability-security provided by the attachment figure to the child, the attachment style developed will be secure (availability and affection) or insecure: preoccupied type (ambivalence, anxiety, fear, and resentment), avoidant type (anger, rejection, and indifference) and traumatized or disorganized type (abuse and lack of affection, alternating between the preoccupied and avoidant styles; Ainsworth, 1991). For its part, resilience refers “to a dynamic process encompassing positive adaptation within the context of significant adversity concept that implies exposure to significant threat and the achievement of positive adaptation despite major assaults on the developmental process” (Luthar et al., 2000, p. 543; also, Connor and Davidson, 2003), while emotional intelligence refers to the ability to manage, discriminate, and use emotions to direct one’s thoughts and actions (Salovey and Mayer, 1990). CPV has also been related to low levels of secure parental attachment (Nowakowski-Sims and Rowe, 2017; Navas-Martínez and Cano-Lozano, 2022b), resilience (Navas-Martínez and Cano-Lozano, 2022b) and emotional intelligence (Margolin and Baucom, 2014; Simmons et al., 2020; Navas-Martínez and Cano-Lozano, 2022b). However, the specific relationship between CPV and the different styles of insecure parental attachment has not been explored to date, although some studies have associated CPV with the lack of parental warmth and parental rejection (Calvete et al., 2015b; Zhang et al., 2019; Cano-Lozano et al., 2020).

More importantly, these variables could act as mediators of the relationship between ACEs and aggressive behavior, as suggested by some studies. For example, insecure attachment mediated the relationship between parental abuse and externalizing symptoms (e.g., aggression and dating violence) in young (Muller et al., 2012; Lee et al., 2013) and adolescents (Moya et al., 2015; Peng et al., 2022); resilience mediated the relationship between ACEs and aggression in a sample of the adult judicial population (Dambacher et al., 2022) and university youth (Chen and Qin, 2020), and low levels of emotional intelligence mediated the relationship between childhood abuse and certain traits associated with violent behavior (callous-unemotional traits) among incarcerated male adolescents with crimes such as injury or theft (Peng et al., 2022). Despite the above, the possible mediators of the relationship between ACEs and CPV have not been analyzed, although the possible mediators of the relationship between parental abuse in particular and CPV have been analyzed, for example, socio-cognitive mechanisms (Contreras et al., 2020; Simmons et al., 2020; Bautista-Aranda et al., 2023; Cano-Lozano et al., 2023). Given the associations between the reviewed variables, it would be convenient to examine whether such relationships take place at different levels, exploring how attachment, resilience, and emotional intelligence intervene in the relationship between ACEs and CPV.

### 1.1. Current study

Based on the literature review, the first objective of this study was to examine the frequency of different types of ACEs and cumulative ACEs in a sample of adolescents who showed CPV compared to adolescents who did not show CPV. We expect to obtain a greater proportion of ACEs in adolescents with CPV than in those without CPV (Calvete et al., 2015a; Contreras and Cano-Lozano, 2016; Simmons et al., 2020; Martín et al., 2022), as well as high percentages of cumulative ACEs in cases of CPV (Navas-Martínez and Cano-Lozano, 2022b).

The second objective was to analyze the differences in parental attachment styles, resilience, and emotional intelligence among CPV aggressors with different levels of cumulative ACEs. The aggressors with cumulative ACEs are expected to be characterized by more insecure parental attachment styles and lower resilience and emotional intelligence than the aggressors without cumulative ACEs; in turn, the same is expected for the aggressors with high levels of cumulative ACEs compared to the aggressors with low levels of cumulative ACEs (Navas-Martínez and Cano-Lozano, 2022b).

**Figure 1:**
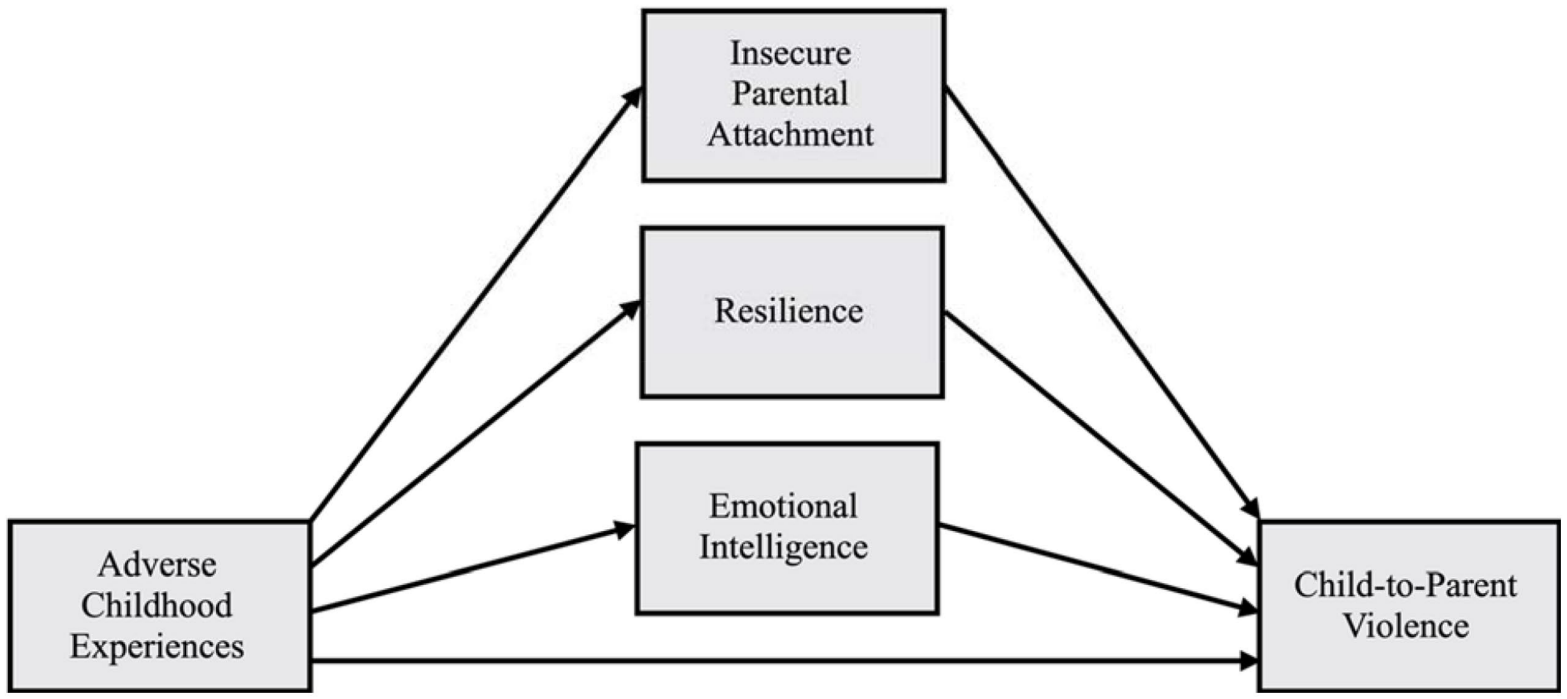
Conceptual mediational model of the relationship between adverse childhood experiences and child-to-parent violence.

The third objective was to examine the associations among the study variables in the group of aggressors with cumulative ACEs. Positive relationships are expected between CPV, ACEs, and insecure parental attachment, as well as negative associations between these variables and resilience and emotional intelligence (Margolin and Baucom, 2014; Nowakowski-Sims and Rowe, 2017; Zhang et al., 2019; Cano-Lozano et al., 2020; Contreras et al., 2020; Navas-Martínez and Cano-Lozano, 2022b). Similarly, we also explored, in the aggressors with cumulative ACEs, the mediating role of insecure parental attachment styles, resilience, and emotional intelligence in the relationship between ACEs and CPV (see Figure 1). Based on studies in the field of violence in general, these variables are expected to mediate this relationship (Moya et al., 2015; Chen and Qin, 2020; Peng et al., 2022).

## 2. Materials and methods

### 2.1. Sample

The sample was formed by 3,142 Spanish (98.3%) adolescents (50.7% females) aged between 12 and 18 years (*M*_age_ = 14.3, *SD* = 1.5) from charter (49.8%) and public (50.2%) educational centers of two regions of Southern Spain. A total of 79.1% lived with both parents and the rest of the participants are from single-parent families and stepfamilies in which they live with one of the parents. Most of the participants informed that they were biological children (98.7%) and from married parents (76.9%).

### 2.2. Instruments

*Child-to-Parent Violence Questionnaire* (*CPV-Q;* Contreras et al., 2019). This instrument evaluates the frequency with which violent behaviors (psychological, physical, financial, and control/domain) are exercised by the adolescent toward the mother (α = 0.75) and the father (α = 0.73) in the last year through 14 parallel items (e.g., “I have hit my parents with something that could hurt them”; “I have told my parents that at home they have to do what I want”) on a Likert scale (0 = *never*; 4 = *very often, six times or more*), with an internal consistency in previous studies between α = 0.79 and α = 0.82 for CPV toward the mother and between α = 0.78 and α = 0.82 for CPV toward the father (Cano-Lozano et al., 2020; Contreras et al., 2020).

*Violence Exposure Scale* (*VES;* Calvete et al., 2014). It evaluates the frequency with which the adolescent experiences violent behaviors (psychological, physical, and verbal) by the mother (direct abuse: α = 0.80) and the father (direct abuse: α = 0.81), as well as the frequency with which the adolescent observes violent behaviors (psychological, physical, and verbal) from the father toward the mother (witness of abuse: α = 0.74), through 6 and 3 items, respectively (e.g., “How many times did your mother insult or humiliate you?”; “How many times did your father hit you?”) on a Likert scale (0 = *never*; 4 = *every day*), with an internal consistency in the original version of α = 0.77 (direct violence subscale) and α = 0.68 (witness of violence subscale; Calvete et al., 2014).

*European Bullying Intervention Project Questionnaire* and *European Cyberbullying Intervention Project Questionnaire* (*EBIP-Q* and *ECIP-Q*; Brighi et al., 2012. Spanish validation; *EBIP-Q*, Del Rey et al., 2012; *ECIP-Q*, Ortega-Ruíz et al., 2016). These two instruments evaluate the frequency with which the adolescent experiences violent behaviors by peers directly (direct abuse: α = 0.81) and online (cyber-abuse: α = 0.74) in the last 2 months through 7 and 11 items, respectively (e.g., “Some has hit me, kicked me, or pushed me”; “Someone has posted compromising videos or photos of me on the internet”) on a Likert scale (0 = *no*; 4 = *yes, more than once a week*), with an internal consistency in the Spanish validation of α = 0.84 (direct violence subscale; Del Rey et al., 2012) and α = 0.80 (cyber-violence subscale; Ortega-Ruíz et al., 2016).

Adverse Childhood Experiences Questionnaire *ad hoc.* This instrument is based on the *ad hoc* Child Adversity Scale (Nowakowski-Sims and Rowe, 2017) used in a CPV sample and on the *Adverse Childhood Experiences International Questionnaire* (*ACE-IQ*; World Health Organization, 2018) which evaluates the presence or absence of a total of 12 adverse experiences related to both parental neglect (parental abandonment) and adverse events experienced by first-person and by family members through 12 dichotomous items (0 = *no*, 1 = *yes,* Ordinal α = 0.94). For example, “I have suffered the abandonment of my mother; “Someone in my family has been in prison for committing a crime.” The Nowakowski-Sims and Rowe (2017) scale shows good internal consistency (Kuder–Richardson 20 binary statistical reliability procedure, KR20 = 0.75), as does the *ACE-IQ* in Spanish (e.g., α = 0.69–0.86; Téllez et al., 2022).

*Attachment Representations Questionnaire* (*CAMIR-R;* Pierrehumbert et al., 1996. Spanish validation; Balluerka et al., 2011). This instrument evaluates past and present experiences of parental attachment, determining a secure or insecure style (preoccupied, avoidant, and traumatized) through 32 items (α = 0.65; e.g., “The idea of a momentary separation from one of my loved ones leaves me with an uneasy feeling”; “Threats of separation, of moving to another place, or of breaking family ties are part of my childhood memories”) on a Likert scale (1 = *strongly disagree*; 5 = *strongly agree*), with an internal consistency in the Spanish validation between α = 0.60 and α = 0.85 (Balluerka et al., 2011).

*Connor and Davidson Resilience Scale* (*CD-RISC-10*; Connor and Davidson, 2003. Spanish validation; Notario-Pacheco et al., 2011). It assesses the degree of adaptation to adverse situations through 10 items (α = 0.81; e.g., “I am able to adapt when changes occur”; “I achieve my goals despite the difficulties”) on a Likert scale (0 = *not at all*, 4 = *almost always*), with an internal consistency in the Spanish validation of α = 85 (Notario-Pacheco et al., 2011).

*Wong and Law Emotional Intelligence Scale* (*WLEIS*; Wong and Law, 2002. Spanish validation; Extremera et al., 2019). This instrument evaluates the level of emotional intelligence through 16 items (α = 0.85; e.g., “I am able to control my own emotions”; “I have a good understanding of the emotions of the people around me”) on a Likert scale (1 = *completely disagree*, 7 = *completely agree*), as well as its different dimensions: intrapersonal emotional perception, or valuation and expression of emotions, interpersonal emotional perception, or valuation and recognition of others’ emotions, emotional assimilation, or use of emotions to facilitate performance, and emotional regulation, or capacity to manage emotions, with an internal consistency in the Spanish validation between α = 0.69 and α = 0.79 (Extremera et al., 2019).

### 2.3. Procedure and design

This is a cross-sectional descriptive study (or *ex post facto*) of populations through surveys (Montero and León, 2007). First, this study was approved by the Ethics Committee of the University of Jaén (Spain; Ref. MAR.18/5.PRY), the Public Administration, and the principals of the participating educational centers. The parents of the adolescent participants received written information about the study and their signed informed consents were obtained. Then, the adolescents who were authorized by their parents to participate in the study were informed and their signed informed consents also were obtained. Participation consisted of the voluntary, anonymous, and confidential completion of a set of paper-and-pencil questionnaires, which were administered by a single evaluator in person and in groups in the classrooms of the educational centers. None of the participants included in the analyses had psychological or developmental disorders or special educational needs.

### 2.4. Data analysis

The internal consistency of the questionnaires was explored with Cronbach’s Alpha coefficient, except for the *ad hoc* Adverse Experiences Questionnaire, whose internal consistency was calculated with the ordinal Alpha coefficient, given the nominal nature of the response scale (Gadermann et al., 2012).

To analyze the frequency of the types of ACEs and cumulative ACEs, we calculated the percentages of adolescents who have experienced behaviors of abuse by parents and peers two or more times (criterion: repeated victimization) and the rest of ACEs (criterion: presence). The differences in such frequencies between the adolescents who have shown CPV behaviors two or more times (criterion: repeated violence) and those who have not were calculated with the χ ^2^ test and the effect size was analyzed with the φ coefficient.

Then, a hierarchical cluster analysis was conducted to explore groups of CPV aggressors with similar levels of cumulative ACEs within each group and different levels of cumulative ACEs between the different groups. Specifically, we used Ward’s clustering method, which measures the squared Euclidean distances (Ward, 1963) maximizing the intragroup homogeneity in the dependent variable (operationalized as the total number of the presence of ACEs, Finkelhor et al., 2007; Pereda et al., 2014). This analysis excluded the aggressors who reported one ACE or none since they did not meet the criterion of accumulation of ACEs. The clusters were verified with a descriptive analysis of the distribution of ACEs. Then, we calculated the differences between the aggressors with different levels of cumulative ACEs and those without cumulative ACEs in the parental attachment styles, resilience, and emotional intelligence through ANOVAs with or without Welch correction and *post hoc* Games-Howell or Bonferroni multiple comparisons according to Leven’s test. The effect sizes of the differences were calculated with Cohen’s *d* statistic test [Cohen, 1988: *d* = 0.20 (small), 0.50 (medium), 0.80 (large)].

Next, we selected the aggressors with cumulative ACEs and performed a correlational analysis to examine the relationships between the study variables. Lastly, multiple mediation analysis was carried out (Hayes, 2017) after verifying that the assumption was fulfilled of significant correlations between (1) predictor and dependent variables, (2) predictor and mediator variables, and (3) mediator and dependent variables. Specifically, we analyzed whether the relationship between ACEs (predictor) and CPV (dependent) is mediated by insecure parental attachment styles, resilience, and emotional intelligence (mediators). The significance of the indirect mediation effects was evaluated with Sobel’s test (Sobel, 1982) and with the confidence intervals generated from the bootstrapping estimation techniques (Preacher and Hayes, 2004). A total of 10,000 bootstraps resamples were used to generate bias corrected 95% CIs for the indirect effects, which were significant when the CI did not contain the value 0.

## 3. Results

Significantly higher proportions of ACEs were found in adolescents who exert CPV compared to those who do not perform this type of violence (see Table 1). For example, 39.8 and 42.3% of aggressors have experienced direct abuse by mother and father, respectively, and 1.2 and 5.7% have experienced abandonment by mother and father, respectively. Peer abuse ranged from 50 to 77% and high percentages of ACEs related to family members were also found (e.g., 63.7% serious physical disease; 51.5% psychological problems). Moreover, the results also show a significantly higher proportion of cumulative ACEs in the group of CPV aggressors (88%) compared to the group of non-CPV adolescents.

**Table 1:** Frequency of the types of adverse childhood experiences and cumulative adverse childhood experiences in child-to-parent aggressors versus non-child-to-parent adolescents.

Adverse childhood experiences^1^	Non-CPV *n* = 1,560	CPV *n* = 1,553	χ ^2^	φ
Direct abuse by mother	343 (22.0)	618 (39.8)	115.9^***^	0.19
Abandonment by mother	8 (0.5)	18 (1.2)	3.9^*^	0.04
Direct abuse by father	373 (23.9)	652 (42.3)	118.6^***^	0.19
Abandonment by father	38 (2.4)	89 (5.7)	21.6^***^	0.08
Witness of abuse father–mother	108 (6.9)	260 (16.7)	71.9^***^	0.15
Direct abuse by peers	1,037 (66.5)	1,198 (77.1)	43.7^***^	0.12
Cyber-abuse by peers	559 (35.8)	791 (50.9)	72.2^***^	0.15
Protection center	4 (0.3)	9 (0.6)	1.9	0.02
Reform center	3 (0.2)	4 (0.3)	0.1	0.00
Serious accident	68 (4.4)	95 (6.1)	4.8^*^	0.04
Serious physical disease	76 (5.3)	94 (6.4)	1.6	0.02
Drug use of family member	133 (8.5)	224 (14.4)	26.6^***^	0.09
Incarceration of family member	74 (4.7)	129 (8.3)	16.2^***^	0.07
Serious accident of family member	515 (33.0)	616 (39.7)	14.9^***^	0.07
Serious physical disease of family member	886 (56.8)	989 (63.7)	15.4^***^	0.07
Psychological problems of family member	585 (37.5)	800 (51.5)	61.9^***^	0.14
Suicide or attempted suicide of family member	68 (4.4)	117 (7.5)	14.0^***^	0.07
Cumulative adverse childhood experiences^2^	1,079 (75.5)	1,277 (88.0)	75.6^***^	0.16

1 Presence of some repeated abuse behavior or some other adverse childhood experience.

2 Presence of two or more adverse childhood experiences.

*n* (%).Non-CPV = group without child-to-parent violence; CPV = group with child-to-parent violence; ^*^p < 0.05. ^***^p < 0.001.

The cluster analysis provided a solution of two CPV aggressors’ groups with different levels of cumulative ACEs. The High-CA group was composed of 274 aggressors (18.9%) with high levels of cumulative ACEs (*M* = 7.9, *SD* = 1.2, range: 7–12) and the Low-CA group was composed of 1,003 aggressors (69.1%) with low levels of cumulative ACEs (*M* = 3.9, *SD* = 1.3, range: 2–6). The third group of the study (Non-CA) was constituted of 174 aggressors (12%) with one ACE or none (*M* = 0.7, *SD* = 0.4, range: 0–1). The intergroup differences in the proportion ( χ ^2^ (24, 1,451) = 2902.0, *p* < 0.001, φ = 1.4) and the mean score of ACEs (*F* = 4732.0, *p* < 0.001) are significant. Furthermore, this score is significantly higher in the High-CA group compared to the Low-CA group (*d* = 3.13) and the Non-CA group (*d* = 7.41) and significantly higher in the Low-CA group compared to the Non-CA group (*d* = 2.64), with excellent effect sizes.

Regarding the analysis of the differences (see Table 2), the two aggressors’ groups with cumulative ACEs (High-CA and Low-CA) obtained lower scores in secure parental attachment and higher scores in insecure parental attachment than the aggressors’ group without cumulative ACEs (Non-CA). In turn, the High-CA group obtained lower scores in secure parental attachment and higher scores in insecure parental attachment than the Low-CA group. Medium and large effect sizes were obtained, with traumatized attachment being the one that best distinguishes the three types of aggressors. The High-CA group obtained lower scores in resilience compared to the Low-CA and Non-CA groups, and no significant differences were identified between the Low-CA and Non-CA groups in this variable. Regarding to emotional intelligence, the two groups with cumulative ACEs obtained lower scores in almost all dimensions compared to the Non-CA, and, in turn, the same was observed for the High-CA group compared to the Low-CA group.

**Table 2:** ANOVAs of the differences between the aggressors with different levels of cumulative adverse childhood experiences and without cumulative adverse childhood experiences.

	High-CA^a^ *n* = 274	Low-CA^b^ *n* = 1,003	Non-CA^c^ *n* = 174	*F*	*Post hoc* comparison (Cohen’s *d*)
PA-Secure	23.6 (4.7)	25.8 (4.2)	27.3 (2.9)	54.1^***^	ab (−0.51), ac (−0.90), bc (−0.37)
PA-Preoccupied	12.5 (3.6)	10.9 (3.4)	9.8 (3.2)	37.0^***^	ab (0.46), ac (0.79), bc (0.33)
PA-Avoidant	13.5 (3.3)	12.0 (3.1)	10.8 (2.7)	45.9^***^	ab (0.48), ac (0.88) bc (0.39)
PA-Traumatized	12.0 (4.4)	9.0 (3.5)	7.4 (2.6)	94.4^***^	ab (0.81), ac (1.21), bc (0.47)
Resilience	24.6 (6.9)	26.3 (6.4)	27.2 (6.2)	10.2^***^	ab (−0.26), ac (−0.39)
EI-Intrapersonal	19.3 (4.9)	20.4 (4.6)	21.4 (4.4)	11.7^***^	ab (−0.24), ac (−0.45), bc (−0.22)
EI-Interpersonal	21.5 (3.9)	21.3 (4.1)	20.9 (3.9)	1.5	n.s.
EI-Assimilation	17.5 (5.6)	19.2 (5.2)	20.5 (4.9)	19.2^***^	ab (−0.32), ac (−0.56), bc (−0.25)
EI-Regulation	15.1 (5.5)	16.7 (5.4)	18.8 (5.5)	24.4^***^	ab (−0.30), ac (−0.67), bc (−0.39)

*M* (*SD*). CA = cumulative adverse childhood experiences; PA = parental attachment; EI = emotional intelligence; ab= significant differences between High-CA group and Low-CA group; ac= significant differences between High-CA group and Non-CA group; bc= significant differences between Low-CA group and Non-CA group; n.s. = not significant. ^***^p < 0.001.

The correlational analysis performed on the aggressors with cumulative ACEs indicates that all variables, except two, are significantly related to each other and in the expected direction (see Table 3). Specifically, CPV is positively related to ACEs. Both variables present positive relationships with insecure parental attachment and negative relationships with secure parental attachment, resilience, and emotional intelligence. Secure parental attachment is positively related to resilience and emotional intelligence, whereas insecure styles, in general, are negatively related to these variables. Lastly, resilience and emotional intelligence present positive relationships between them.

**Table 3:** Correlational analysis between the study variables.

	1	2	3	4	5	6	7	8	9	10
CPV	–									
ACEs	0.36^***^	–								
PA-Secure	−0.15^***^	−0.33^***^	–							
PA-Preoccupied	0.18^***^	0.31^***^	−0.24^***^	–						
PA-Avoidant	0.16^***^	0.30^***^	−0.37^***^	0.32^***^	–					
PA-Traumatized	0.20^***^	0.38^***^	−0.43^***^	0.31^***^	0.35^***^	–				
Resilience	−0.09^**^	−0.18^***^	0.21^***^	−0.09^**^	−0.02	−0.08^**^	–			
EI-Intrapersonal	−0.07^*^	−0.13^***^	0.25^***^	−0.05	−0.12^***^	−0.15^***^	0.39^***^	–		
EI-Assimilation	−0.10^**^	−0.19^***^	0.27^***^	−0.12^***^	−0.15^***^	−0.12^***^	0.53^***^	0.37^***^	–	
EI-Regulation	−0.21^***^	−0.24^***^	0.17^***^	−0.12^***^	−0.11^***^	−0.15^***^	0.48^***^	0.49^***^	0.34^***^	–

*N* = 1,277. CPV = child-to-parent violence; ACEs = adverse childhood experiences; PA = parental attachment; EI = emotional intelligence. ^*^p < 0.05. ^**^p < 0.01. ^***^p < 0.001.

**Figure 2:**
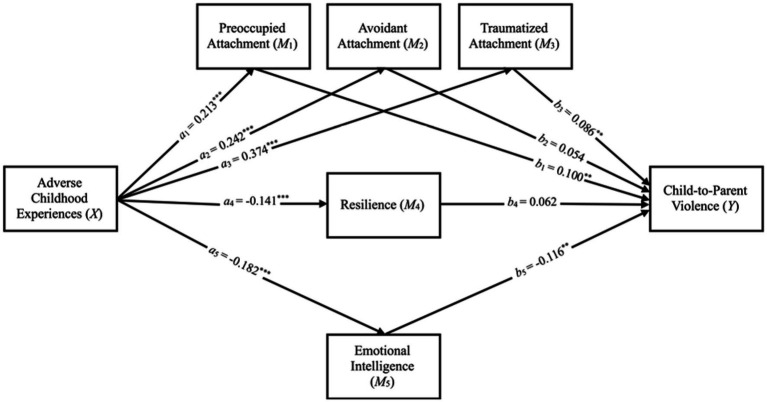
Statistical mediational model of the relationship between adverse childhood experiences and child-to-parent violence. *X* = predictor variable; *M* = mediator variable; *Y* = dependent variable. *c’* = 0.207, *p* < 0.001; *c* = 0.286, *p* < 0.001. ^**^ *p* < 0.01. ^***^ *p* < 0.001.

Finally, the proposed mediation model for CPV was tested. Figure 2 represents the results of the effects of the model components, as well as the total and direct effects, both statistically significant, suggesting a partial mediation effect.

Specifically, Table 4 shows that the total effect of the relationship between ACEs and CPV is significant. This total effect of this relationship is due to the direct relationship effect between these variables as well as the indirect relationship effect through preoccupied and traumatized attachment and also through low emotional intelligence.

**Table 4:** Mediation effects of adverse childhood experiences on child-to-parent violence.

Effects	Path	Coeff.	*B*	*SE*	*t*/*Z*	*p*	LLCI, ULCI^1^
Total	ACEs → PA → Resilience → EI → CPV	*c*	0.286	0.116	10.597	< 0.001	0.999, 1.453
	*R*^2^ = 0.082, *F* = 112.301, *p* < 0.001						
Direct	ACEs → CPV	*c′*	0.207	0.125	7.116	< 0.001	0.643, 1.133
Indirect	ACEs → PA-Preoccupied → CPV	*a*_1_ * *b*_1_	0.021	0.007	3.165	0.001	0.008, 0.036
	ACEs → PA-Avoidant → CPV	*a*_2_ * *b*_2_	0.013	0.008	1.781	0.074	−0.002, 0.029
	ACEs → PA-Traumatized → CPV	*a*_3_ * *b*_3_	0.032	0.013	2.799	0.005	0.006, 0.059
	ACEs → Resilience → CPV	*a*_4_ * *b*_4_	−0.009	0.006	−1.735	0.082	−0.021, 0.002
	ACEs → EI → CPV	*a*_5_ * *b*_5_	0.021	0.008	3.090	0.002	0.007, 0.037

1 Bootstrapping results with confidence intervals for the lower (LLCI) and upper limits (ULCI).

*N* = 1,277. Coeff. = coefficients; ACEs = adverse childhood experiences; PA = parental attachment; EI = emotional intelligence; CPV = child-to-parent violence.

## 4. Discussion

The aim of this study was to analyze CPV from the framework of adverse childhood experiences (ACEs), focusing on cumulative ACEs. Regarding the first objective, it was found that ACEs, both independently and cumulatively, were more frequent in the adolescents who exert CPV with respect to those who did not perform this type of violence, thus confirming the proposed hypotheses.

This study found that the ACEs related to abuse were more frequent in adolescents with CPV than in those without CPV, which is in line with previous research (Contreras and Cano-Lozano, 2016; Simmons et al., 2020; Martín et al., 2022). Specifically, regarding ACEs related to parental victimization, 39.8% suffered abuse by the mother, 42.3% suffered abuse by the father, and 16.7% witnessed abuse from the father toward the mother. Nowakowski-Sims and Rowe (2017) also found high percentages of these ACEs in adolescents with CPV offenses (25% direct parental abuse and 54% witnessing of parental abuse), although the percentages of both types of abuse are different from those obtained in the present study. This could be due to the fact that the authors do not distinguish between mothers and fathers, which suggests the need to evaluate these ACEs taking into account the complexity they show in real life. In this sense, it is also necessary to evaluate in future studies the exposure to violence from the mother toward the father. On the other hand, Nowakowski-Sims and Rowe (2017), find that 48% of adolescents with CPV offenses reported having uninvolved parents, while this study goes deeper in this direction finding that 1.2 and 5.7% of CPV aggressors reported abandonment by the mother and father, respectively, with abandonment percentages likely to be higher in judicial population. With regard to ACEs related to peer victimization, the results showed that 77.1% of CPV aggressors suffered direct abuse by peers, which is in line with the findings of a previous study where most of the aggressors suffered this type of abuse (Calvete et al., 2015a). Moreover, 50.9% suffered cyber-abuse by peers, which provides new data in CPV cases. The other types of ACEs were also more frequent in adolescents with CPV than in those without CPV, providing information that had not been analyzed to date.

Globally, there were high rates of ACEs in adolescents who exerted CPV. Specifically, 96.6% had at least one ACE and 88% had an accumulation of two or more of them, thus confirming the hypothesis that established high rates of cumulative ACEs in the sample of CPV aggressors (Navas-Martínez and Cano-Lozano, 2022b). The mentioned study found that 22% of CPV aggressors experienced cumulative victimization (parental and peer abuse), which, compared with 88% found in this study, could suggest the relevance of evaluating the presence of accumulation of other ACEs, in addition to those related to abuse. We also found that the proportion of cumulative ACEs was significantly higher in adolescents with CPV than in those without CPV; although these findings cannot be compared with those of previous studies, due to the absence of the latter, they provide novel data to the field of CPV.

Based on the conclusions of the study of aggressors’ profiles (Moulds et al., 2019; Nowakowski-Sims, 2019; Martínez-Ferrer et al., 2020; Vecina et al., 2020; Boxall and Sabol, 2021; Navas-Martínez and Cano-Lozano, 2022b,c, 2023; Cano-Lozano et al., 2023), the second objective was to examine the differences in the parental attachment styles, resilience, and emotional intelligence between CPV aggressors with different levels of cumulative ACEs and without cumulative ACEs Specifically, from the 88% of aggressors who had two or more ACEs, 69.1% showed low levels (between 2 and 6 ACEs) and 18.9% showed high levels (between 7 and 12 ACEs; Finkelhor et al., 2007; Pereda et al., 2014). The other 12% of aggressors had one ACE or none.

It was found that aggressors with cumulative ACEs in general were characterized by high levels of insecure parental attachment, lower levels of resilience, and lower emotional intelligence compared to those without cumulative ACEs. These results are consistent with those of Navas-Martínez and Cano-Lozano (2022b), who found that CPV aggressors with victimization experiences present less parental attachment and less resilience and emotional competencies than aggressors without victimization experiences. Moreover, our data improve the understanding of this profile, with the addition of another type of ACEs, which also seems to be frequent and more usual in adolescents who exerted CPV than in those who did not exert this type of violence, and delving into the different styles of insecure parental attachment. Furthermore, and more importantly, it was observed that, with the increase in the number of ACEs, there was a decrease in the levels of parental security, resilience, and emotional competencies of the aggressors. Specifically, those with high cumulative adversity levels were characterized by a more insecure parental attachment, lower resilience, and lower emotional intelligence than those with low cumulative adversity levels. Again, these results are in agreement with those of Navas-Martínez and Cano-Lozano (2022b), who found more affected areas in CPV aggressors with more types of abuse than in those with a single type. In addition, another finding of this study shows that only aggressors with high levels of cumulative adversity are characterized by less resilience compared to aggressors without cumulative adversity and those with low levels of cumulative adversity, showing these latter similar levels of resilience. The results might suggest that, although having resilience might be helpful in adapting to adverse situations, the large number of ACEs might reduce the capacity to adapt adequately. In fact, some studies indicate that the higher the level of ACEs, the lower the resilience capacity (e.g., Arslan, 2016), and the associations obtained in this study also confirm these findings. However, more studies on CPV are needed to further clarify the role of resilience.

Furthermore, particularly notable differences were identified between the three groups of aggressors in traumatized attachment and in emotional assimilation and regulation. This could indicate that aggressors with cumulative ACEs, especially those with higher levels, tend to develop mental representations of their parents, themselves, and the world around them characterized by abuse and the lack of affection, as well as serious difficulties in the use and regulation of their own emotions compared to the aggressors without cumulative ACEs. Our results support previous studies which point out that simultaneous exposure to multiple types of abuse is a powerful indicator of socio-emotional functioning deterioration in adolescents (Finkelhor et al., 2013; Lätsch et al., 2017; Barnett and Howe, 2021) and in cases of CPV (Navas-Martínez and Cano-Lozano, 2022b), and they also expand these results by adding a more complete set of ACEs and analyzing their greater or lesser levels of accumulation.

The results also confirm the hypothesis related to the third objective. The correlational analysis showed, in the profile of aggressors with cumulative adversity, positive and significant associations between CPV and ACEs, which would support the results of previous studies that report associations between CPV and parental and peer abuse (e.g., Margolin and Baucom, 2014; Beckmann, 2019; Contreras et al., 2020; Simmons et al., 2020; Cano-Lozano et al., 2021b; Navas-Martínez and Cano-Lozano, 2022a), and it could suggest that CPV is also related to a wider range of ACEs. Likewise, in the line of previous studies, the results also showed that CPV was negatively associated with secure parental attachment (Nowakowski-Sims and Rowe, 2017; Navas-Martínez and Cano-Lozano, 2022b), and positively associated with all insecure attachment styles, which supports the results of studies that analyze similar variables (Calvete et al., 2015b; Zhang et al., 2019; Cano-Lozano et al., 2020) and provides novel data in cases of CPV. CPV was also negatively related to resilience (Navas-Martínez and Cano-Lozano, 2022b) and emotional intelligence (Margolin and Baucom, 2014; Simmons et al., 2020; Navas-Martínez and Cano-Lozano, 2022b).

Moreover, the results showed that ACEs directly predicted CPV. No study about CPV had analyzed the predictive capacity of a wide range of ACEs to date. From our results, it would be interesting to analyze the role of each type of adversity separately. Likewise, this study explored the mediating role of some of the variables linked in the literature to both CPV and ACEs as a first step to understanding the explanatory mechanisms through which ACEs are related to CPV. This is supported by previous studies about CPV (Contreras et al., 2020; Simmons et al., 2020; Navas-Martínez and Cano-Lozano, 2022b; Cano-Lozano et al., 2023) and about the field of youth violence in general (Moya et al., 2015; Chen and Qin, 2020; Peng et al., 2022), which suggests that the variables explored in this study could operate at different levels. Specifically, we found that the relationship between ACEs and CPV is best explained through the effect of preoccupied and traumatized attachment and through low levels of emotional intelligence, suggesting that childhood adversity experiences could influence the development of mental representations of emotional bonding with parents characterized by anxiety, fear, abuse, and lack of affection, as well as the development of less emotional competencies, which in turn would trigger CPV. However, unlike previous studies found that resilience mediated the relationship between ACEs and aggression in a sample of the adult judicial population (Dambacher et al., 2022) and university youth (Chen and Qin, 2020), in this study resilience did not mediate the relationship between ACEs and CPV, suggesting the need for more research on CPV focusing on the role of resilience in these aggressors.

In summary, the present study found higher rates of a variety of ACEs, both independently and cumulatively, as well as a greater frequency of all of them in adolescents who exerted CPV than in those who did not perform this type of violence. More specifically, 88% of the aggressors showed an accumulation of several ACEs and were identified a profile of aggressors with cumulative ACEs, including those with high and low levels of cumulative ACEs. In general, the aggressors of this profile had more insecure parental attachment styles, lower resilience, and lower emotional intelligence than the aggressors who did not present cumulative adversity. Furthermore, those with high levels of cumulative adversity were characterized by more insecure parental attachment styles, lower resilience, and lower emotional intelligence than the aggressors with low levels of cumulative adversity. Finally, ACEs were related to CPV through preoccupied and traumatized parental attachment and also through low levels of emotional intelligence.

This study has some limitations that must be pointed out. Firstly, it was not possible to establish causality, given the cross-sectional nature of the data. Secondly, the results are extracted from the self-report of the adolescents and the conclusions are based on a representative sample of Southern Spain, which limits the generalization of the results to other populations. Thirdly, a larger number of family ACEs were evaluated compared to extra-family ACEs. Therefore, to address these limitations, it would be convenient that future longitudinal studies replicate this study in other regions of Spain and in other countries, evaluating a larger number of ACEs (sibling and dating abuse, witnessing of sibling, dating, peer and mother-to-father abuse) and using a larger number of self-reports (parents, siblings, partners, and peers), in addition to that of adolescents.

Despite these limitations, it is considered that the findings of this study have some important implications for research and professional practice. In the case of research, the results suggest the convenience of delving into the profiles of aggressors with ACEs, exploring a larger number of characteristics and comprising more areas, such as social. In the long term, the study of aggressors’ profiles could contribute to the development of as many specialized interventions as profiles identified in the literature. It would also be interesting to test the model analyzed in this study according to the type of ACE and verify whether the same effects are obtained in the case of CPV toward mothers and fathers, or whether different effects are observed. Regarding professional practice, the differences found in this study in different types of aggressors suggest the need to redesign the interventions on CPV aimed at covering the most deteriorated areas in each specific case (e.g., resilience in the aggressors with high levels of accumulation of ACEs). In the case of professional practice, it would be important to work with children exposed to ACEs because of the repercussions that ACEs could have on the development of mental representations of negative bonds and emotional difficulties. Likewise, interventions on CPV with children who have experienced certain types of ACEs could focus on improving attachment style and training emotional competencies, whereas when negative attachments have been established with violent parents it would be useful to work with children in the construction of new mental representations of healthy relationship models towards other significant attachment figures, in order to help them establish new positive bonds, in addition to improving their emotional competencies.

In conclusion, although further research is necessary, this study provides a better understanding of child-to-parent violence from the perspective of adverse childhood experiences, especially of those cases that involve an accumulation of adverse experiences during childhood, and it suggests the convenience of greater professional attention to this type of cases with the design of specialized CPV intervention programs.

## Data availability statement

The raw data supporting the conclusions of this article will be made available by the authors, without undue reservation.

## Ethics statement

This study, which involves human participants, was reviewed and approved by Ethic Committee of the University of Jaén. Written informed consent to participate in this study was provided by the participants’ legal guardian/next of kin.

## Author contributions

MN-M and MC-L contributed to conceptualization, methodology, validation, data curation, writing original draft preparation, review, and editing, and funding acquisition. MN-M performed the statistical analysis. MC-L supervised the study. All authors contributed to the article and approved the submitted version.

## Funding

This study was funded by a Predoctoral Fellowship for Research Personnel Development of the University of Jaén [grant number R6/04/2018].

## Conflict of interest

The authors declare that the research was conducted in the absence of any commercial or financial relationships that could be construed as a potential conflict of interest.

## Publisher’s note

All claims expressed in this article are solely those of the authors and do not necessarily represent those of their affiliated organizations, or those of the publisher, the editors and the reviewers. Any product that may be evaluated in this article, or claim that may be made by its manufacturer, is not guaranteed or endorsed by the publisher.
